# Night and day variations of sleep in patients with disorders of consciousness

**DOI:** 10.1038/s41598-017-00323-4

**Published:** 2017-03-21

**Authors:** Malgorzata Wislowska, Renata del Giudice, Julia Lechinger, Tomasz Wielek, Dominik P. J. Heib, Alain Pitiot, Gerald Pichler, Gabriele Michitsch, Johann Donis, Manuel Schabus

**Affiliations:** 1 Laboratory for Sleep, Cognition and Consciousness, & Centre for Cognitive Neuroscience, University of Salzburg, Salzburg, Austria; 2 Laboratory of Image & Data Analysis, Ilixa Ltd., Nottingham, United Kingdom; 3 Apallic Care Unit, Neurological Division, Albert-Schweitzer-Klinik, Graz Austria; 4 Apallic Care Unit, Neurological Division, Pflegewohnhaus Donaustadt, Vienna Austria

## Abstract

Brain injuries substantially change the entire landscape of oscillatory dynamics and render detection of typical sleep patterns difficult. Yet, sleep is characterized not only by specific EEG waveforms, but also by its circadian organization. In the present study we investigated whether brain dynamics of patients with disorders of consciousness systematically change between day and night. We recorded ~24 h EEG at the bedside of 18 patients diagnosed to be vigilant but unaware (Unresponsive Wakefulness Syndrome) and 17 patients revealing signs of fluctuating consciousness (Minimally Conscious State). The day-to-night changes in (i) spectral power, (ii) sleep-specific oscillatory patterns and (iii) signal complexity were analyzed and compared to 26 healthy control subjects. Surprisingly, the prevalence of sleep spindles and slow waves did not systematically vary between day and night in patients, whereas day-night changes in EEG power spectra and signal complexity were revealed in minimally conscious but not unaware patients.

## Introduction

**Figure 1:**
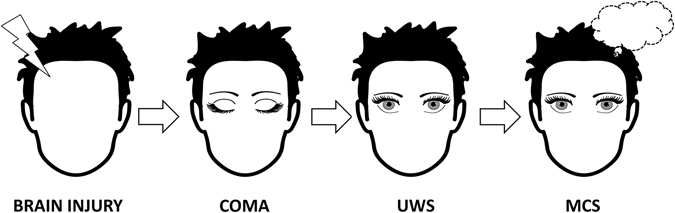
Recovery after severe brain injury. According to the clinical definition, transition from coma to Unresponsive Wakefulness Syndrome (UWS) is denoted by the eyes opening, which is interpreted as a sign of regained arousal. Reproducible evidence of recovered awareness is a hallmark of Minimally Conscious State (MCS), but is already assigned as soon as a patient can visually fixate (MCS−). A step further up, patients are termed MCS+ if they are sometimes able to communicate in some form with the environment.

When it comes to classifying awareness it is particularly deceiving to rely on pure observation as an individuum can be well aware of his environment with closed eyes or completely unaware even though his eyes are open, as believed in some post-comatose states. Furthermore, it is well known that severe brain injuries deteriorate brain dynamics and can lead to impairment of consciousness. Survivors, who awake from coma and show periods of eye opening and closing, seem to exhibit fluctuations of arousal that resemble circadian sleep-wake cycling of healthy individuals. This putative manifestation of vigilance is also a hallmark for the clinical diagnosis of the Unresponsive Wakefulness Syndrome^1^ (UWS; cf. Fig. 1). When in addition to signs of regained arousal, also reproducible evidence of awareness is detected, a patient is diagnosed to be in a Minimally Conscious State (MCS; introduced by Giacino in 2002)^2^. In a clinical setting the assessment of subjects with Disorders of Consciousness (DOC) is based on the observation of overt behavior, a measure that is known to be problematic as behavioral signs are often ambiguous in this patient population^3^.

According to a prevalent theory consciousness is related to the brain’s ability to integrate information through efficient neural networks^4–6^. Inter-regional connectivity underlies also the generation of sleep-specific oscillatory patterns, like sleep spindles^7^. Consciousness levels usually also vary across day and night with conscious awareness prevailing during the day and fading during sleep. Similarly, consciousness gradually changes during the course of DOC recovery. These states of altered awareness are characterized by systematic changes in oscillatory brain dynamics in the healthy and lesioned brain^8,9^. Interestingly, patients with capability of covert command following in the fMRI were found to have preserved electroencephalographic (EEG) organization across sleep and wakefulness including sleep spindling^10^. This intricate relationship between consciousness, circadian rhythmicity, neural connectivity and brain oscillations constitutes an intriguing and compelling puzzle which may help to shed some light on the biggest mystery in humans, their fluctuating conscious awareness of themselves and their surrounding world.

Interestingly the study of sleep-wake alterations and sleep architecture in DOC patients have hardly been investigated, yet constitute a promising way to better understand the association between behavioral and neural signs of arousal, and most importantly, the structural and functional integrity of neural networks. Discoveries in this field therefore should have further relevance for diagnosis, prognosis^11^ and also the more general understanding of the neural correlates of consciousness^12^.

Landsness and colleagues were among the first to describe sleep and especially the homeostatic regulation of slow wave activity in UWS and MCS patients^13^. Furthermore their results suggested that patients’ clinical state was associated to the clear-cut presence (i.e. MCS patients) or absence (i.e. UWS patients) of rapid/non-rapid eye movement sleep stage (REM/nonREM) cycles, and specific electrophysiological sleep features such as sleep spindles and slow waves. Yet it has to be mentioned that following research reports drew a less straightforward picture^10,14–18^.

We found sleep staging of DOC patients with their often highly altered EEG (as well as EMG and EOG) activity rather problematic and impossible to realize when applying accepted sleep staging criteria. Instead, we decided to opt for a more data-driven approach and investigated whether patients fluctuate systematically between light-on day and light-off night periods across 24 hours in their oscillatory EEG activity. We hypothesised that the difference between the clinical entities UWS and MCS are reflected in variations of brain dynamics which can be observed at bedside across day and night. It was expected that light as one of the most potent “Zeitgebers” in humans, would systematically alter brain dynamics between day and night periods even in the absence of conscious awareness.

## Results

For the purpose of assessing diurnal variations in oscillatory activity in different DOC states, we recorded continuous, long term (~24 h) polysomnography (PSG) in a sample of 35 patients. The recordings were then divided into periods of lightsomeness, which corresponds to circadian day (named day-time), and periods of darkness, which corresponds to circadian night (named night-time). For comparison, overnight (~8 h) recordings of 26 healthy control subjects were sleep staged and divided into sleep (analogously named “night-time”) and wake periods (analogously termed “day-time” in the following). Day-night differences in (i) spectral power, (ii) sleep-specific oscillatory grapho-elements, and (iii) EEG signal complexity were investigated, with a special focus on interaction between diurnal changes in brain activity (DIURNAL TIME: day-time, night-time) and varying level of consciousness as reflected by the clinical diagnoses (DIAGNOSIS: UWS, MCS, control).

### Power Spectral Density Estimation

We observed that diurnal variations of EEG oscillatory power distribution differed across the investigated groups. We utilized two ratios (high 8–30 Hz to low 1–8 Hz; as well as alpha to theta), which were previously found to be robustly associated in the patient population with coma recovery scale-revised (CRS-R) scores^19^, a diagnostic tool where the higher score reflects better clinical condition. We found diurnal fluctuations of frequency power ratios to be associated with the behaviorally evaluated level of consciousness.

The analyses revealed significant DIURNAL TIME × DIAGNOSIS interaction in alpha-to-theta frequency ratio and in high-to-low frequency ratio over all three assessed cortical sites. Specifically, the alpha-to-theta ratio revealed highly significant interactions over frontal (*F*_2,56_ = 15.460, *p* < 0.001, ${\eta }_{p}^{2}=0.356$), central (*F*_2,54_ = 24.533, *p* < 0.001, ${\eta }_{p}^{2}=0.476$) and parietal (*F*_2,54_ = 29.634, *p* < 0.001, ${\eta }_{p}^{2}=0.523$) midline cortical sites. Similarly, the interaction term for high-to-low frequency ratio was significant for frontal (*F*_2,56_ = 17.898, *p* < 0.001, ${\eta }_{p}^{2}=0.390$), central (*F*_2,54_ = 31.839, *p* < 0.001, ${\eta }_{p}^{2}=0.541$) and parietal (*F*_2,54_ = 25.240, *p* < 0.001, ${\eta }_{p}^{2}=0.483$) midline cortical sites.

**Figure 2:**
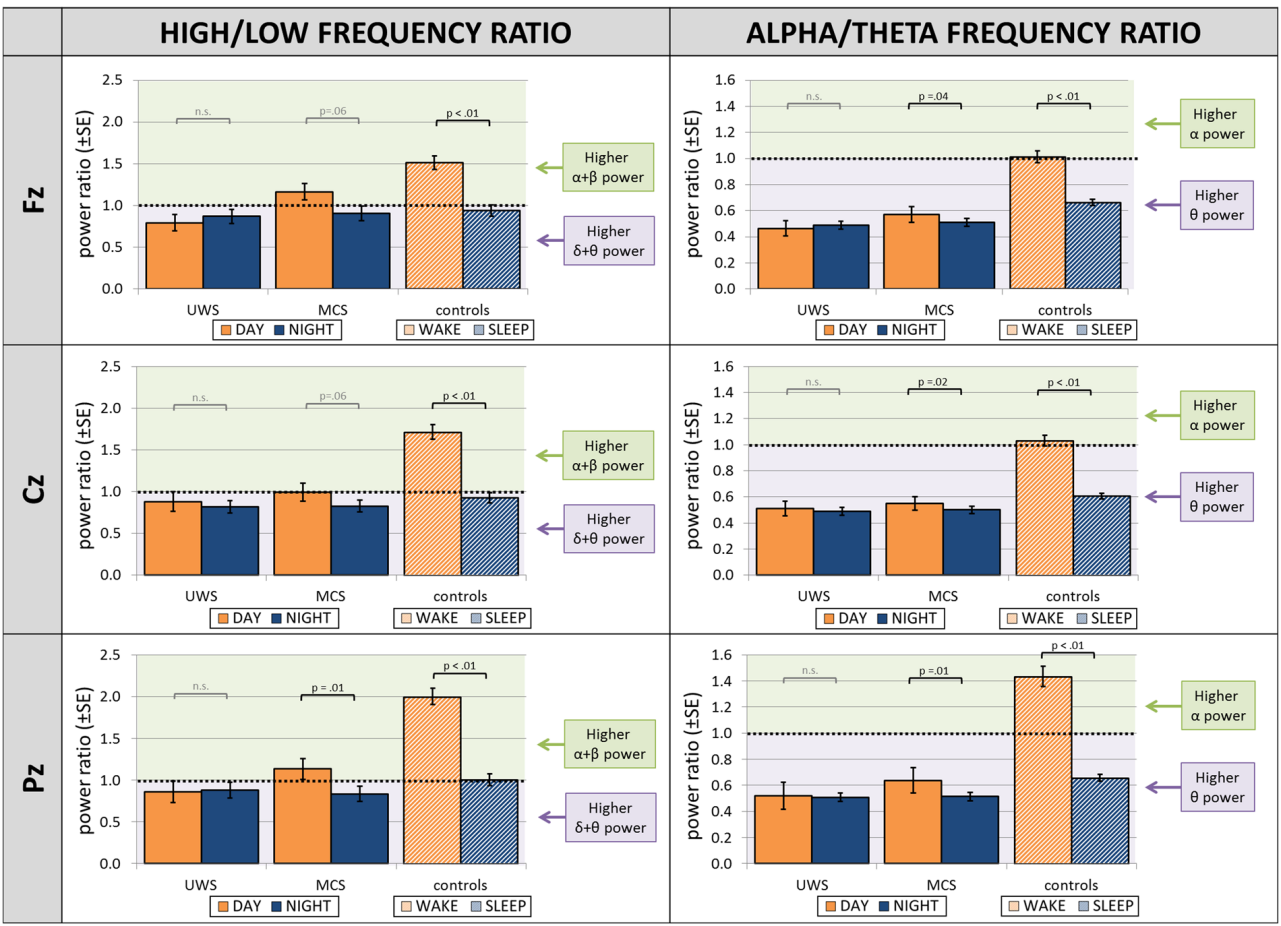
Power spectra ratios across day and night. Both MCS and control subjects revealed statistically significant changes from day to night-time of high-to-low and of alpha-to-theta frequency power ratios, indicating a prevalence of higher frequencies during day-time. No diurnal variation in these measures was observed in UWS patients. High frequencies range from 8–30 Hz, low frequencies range from 2–8 Hz, alpha is defined as 8–12 Hz and theta as 4–8 Hz. Abbreviations: UWS = Unresponsive Wakefulness Syndrome; MCS = Minimally Conscious State.

High-to-low and alpha-to-theta frequency power ratios were significantly higher during day compared to night-time for all recordings sites in the control group and (marginally) significant in the MCS group. In UWS, no significant results were observed (see Fig. 2). Specifically, MCS patients had larger high-to-low frequency power ratio during day-time than during night-time over parietal midline electrode (*t*_15_ = 2.066, *p* = 0.011, *r* = 0.662), and revealed a trend towards statistical significance over frontal (*t*_15_ = 2.274, *p* = 0.055, *r* = 0.506) and central (*t*_16_ = 2.066, *p* = 0.055, *r* = 0.459) midline electrodes. In controls the difference was statistically signifianct for all three cortical sites (Fz: *t*_25_ = 9.035, *p* < 0.001, *r* = 0.875; Cz: *t*_25_ = 8.898, *p* < 0.001, *r* = 0.872; Pz: *t*_25_ = 9.437, *p* < 0.001, *r* = 0.884). The alpha-theta ratio was significantly larger during day-time compared to night-time in MCS over all three cortical sites (Fz: *t*_15_ = 2.248, *p* = 0.040, *r* = 0.502; Cz: *t*_16_ = 2.824, *p* = 0.012, *r* = 0.577; Pz: *t*_15_ = 3.335, *p* = 0.005, *r* = 0.653). Also in control subjects the difference was robust and statistically significant for all three cortical sites (Fz: *t*_25_ = 5.892, *p* < 0.001, *r* = 0.763; Cz: *t*_25_ = 8.373, *p* < 0.001, *r* = 0.859; Pz: *t*_25_ = 7.346, *p* = 0.002, *r* = 0.827).

Between-group comparison of effect sizes revealed significantly stronger day-to-night-time change in control compared to MCS sample for high-to-low ratio over all three cortical sites (Fz: *z*_*diff*_ = 2.29, *p* = 0.001, one-tailed; Cz: *z*_*diff*_ = 2.49, *p* = 0.006, one-tailed; Pz: *z*_*diff*_ = 1.71, *p* = 0.004, one-tailed) and for alpha-to-theta ratio over Cz (*z*_*diff*_ = 1.86, *p* = 0.031, one-tailed). Effect size was significantly higher for MCS compared to UWS for high-to-low ratio over Fz (*z*_*diff*_ = 1.46, *p* = 0.042, one-tailed) and Pz (*z*_*diff*_ = 2.02, *p* = 0.022, one-tailed), as well as by trend for alpha-to-theta ratio over Fz (*z*_*diff*_ = 1.63, *p* = 0.052, one-tailed). For a detailed description of diurnal changes of each of the frequency bands separately, please refer to the Supplementary Material (Suppl. Fig. 1).

### Oscillatory Grapho-Elements During Sleep: Sleep Spindles and Slow Waves

**Figure 3:**
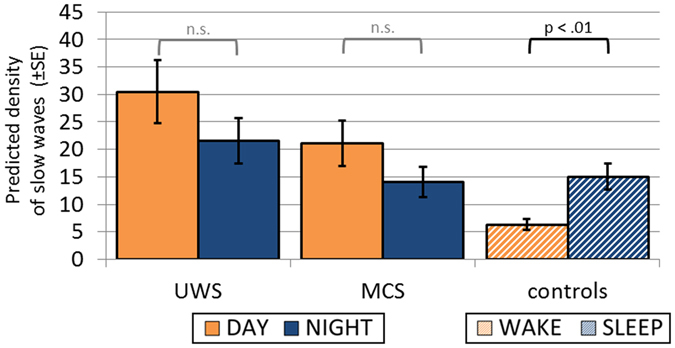
Frequency of occurrence of slow waves across day and night. Note that although liberal detection criteria were applied, the number of detected slow waves was significantly larger during night than day-time in controls only. Abbreviations: UWS = Unresponsive Wakefulness Syndrome; MCS = Minimally Conscious State.

Further we explored diurnal changes in the frequency of occurrences of sleep specific oscillatory patterns: sleep spindles (over frontal, central and parietal sites) and slow waves (over frontal regions). We modelled the relationships between the likelihood of occurrence of these oscillatory sleep patterns on the one hand, and DIAGNOSIS as well as DIURNAL TIME on the other hand. The negative binomial models were significant for sleep spindles in the frontal (*χ* ^2^(5) = 19.152, *p* = 0.002; AIC = 1221), central (*χ* ^2^(5) = 62.528, *p* < 0.001; AIC = 1115) and parietal (*χ* ^2^(5) = 65.934, *p* < 0.001; AIC = 1190) poles, as well as for slow waves (*χ* ^2^(5) = 39.253, *p* < 0.001; AIC = 2226). However, the DIAGNOSIS × DIURNAL TIME interaction turned out to be a significant predictor only for number of frontal slow waves (*Wald χ* ^2^(2) = 17.900, *p* < 0.001) (Fig. 3). The number of predicted slow waves (*Wald χ* ^2^(1) = 11.511, *e* ^*β*^ = 2.38, *p* = 0.001) was significantly different for day and night-time only in the control group, suggesting the prevalence of this EEG sleep-specific pattern during the night in the sample of healthy subjects.

**Figure 4:**
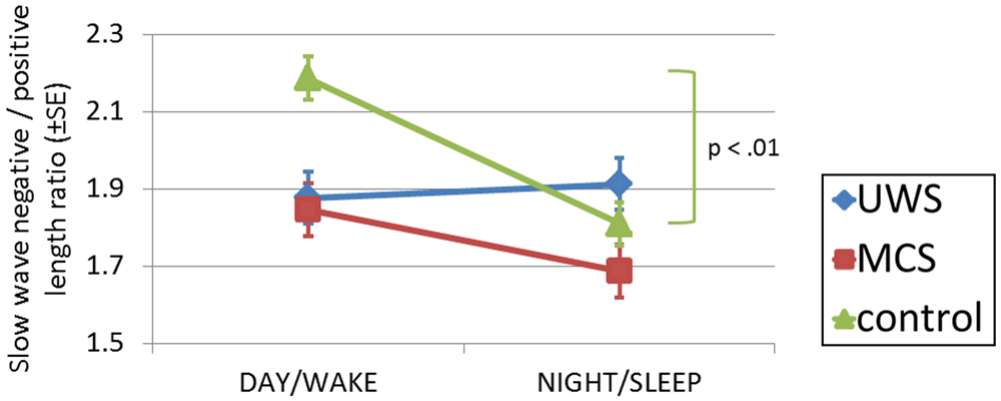
Morphology of detected slow waves. Slow-waves were more symmetrical during night than day-time in controls only. A similar day to night-time change was observed in MCS patients; however the difference did not reach the level of statistical significance. Abbreviations: UWS = Unresponsive Wakefulness Syndrome; MCS = Minimally Conscious State.

We also had a closer look at the morphology of the detected slow waves, and computed their peak-to-peak amplitude (measure of magnitude) and ratio of the positive to negative slow wave length (measure of asymmetry). We observed significant DIAGNOSIS × DIURNAL TIME interaction for the asymmetry ratio (*F*_2,58_ = 10.901, *p* < 0.001, ${\eta }_{p}^{2}=0.273$). Follow-up analysis revealed a significant difference between day and night-time solely in the healthy control group, with more symmetrical slow waves (*t*_25_ = 5.949, *p* < 0.001, *r* = 0.766, FDR-corrected) during the night-time (Fig. 4). The grand-average slow waves for both clinical entities and day-night can be seen in Suppl. Fig. S2.

**Figure 5:**
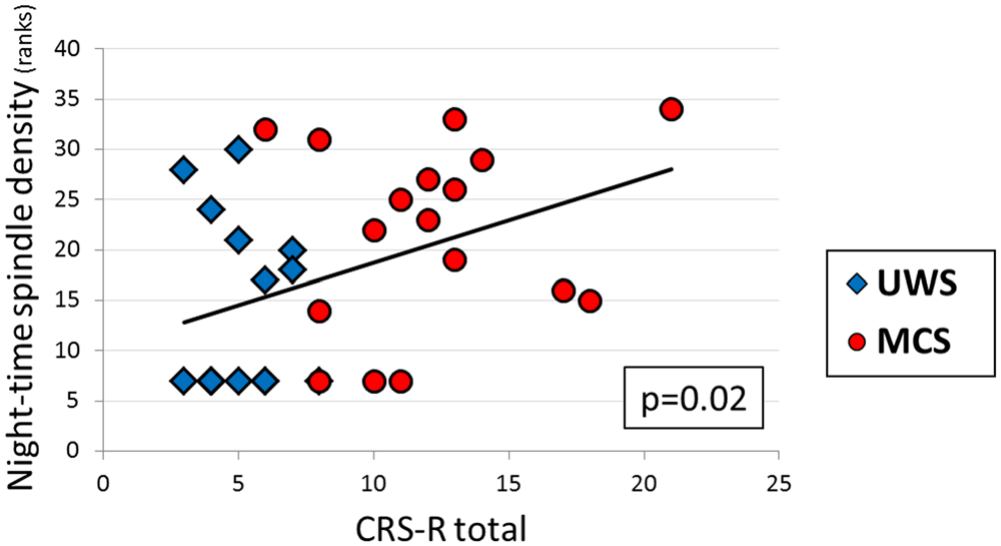
Correlations between density of parietal sleep spindles and total CRS-R scores. The more parietal spindles during night-time the higher CRS-R scores. Each dot on the graph corresponds to one patient (N = 34). Correlations were calculated for ranked values. Abbreviation: CRS-R = Coma Recovery Scale-Revised.

Furthermore, we investigated the association between the behavioural patient’s state (CRS-R) and the density of sleep specific grapho-elements. We observed a statistically significant positive correlation (*r*_*s*_ = 0.40, *p* = 0.020) between the CRS-R score and the density of parietal sleep spindles during night-time (Fig. 5). Interestingly, the CRS-R score was also negatively correlated with the amount of slow waves, but only during the night-time (*r*_*s*_ = −0.33, *p* = 0.060).

### Permutation Entropy

**Figure 6:**
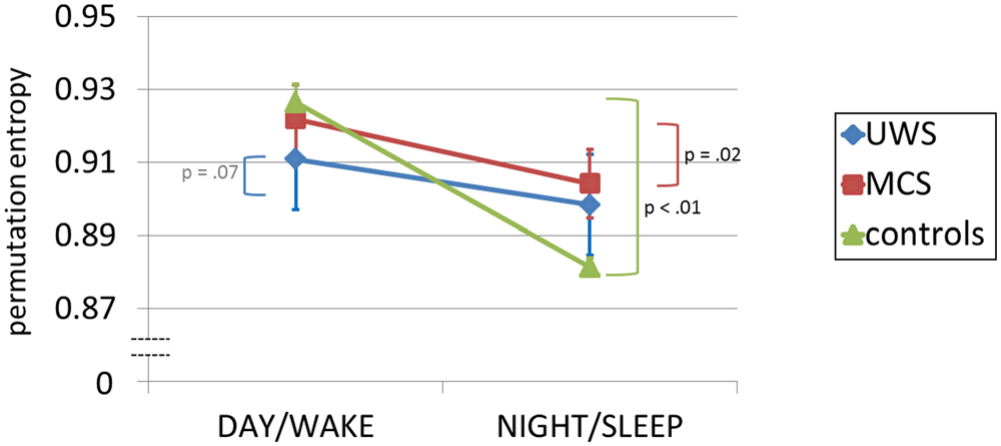
EEG complexity change from day to night averaged across the whole brain. Note that signal complexity decreases significantly from day to night in control and MCS patients. In UWS patients we could observe a trend towards a significant change. Abbreviations: PE = Permutation Entropy, UWS = Unresponsive Wakefulness Syndrome, MCS = Minimally Conscious State.

The complexity of the EEG signal was investigated with permutation entropy (PE) measure, and revealed significant DIURNAL TIME × DIAGNOSIS interaction (*F*_2,58_ = 9.400, *p* < 0.001, ${\eta }_{p}^{2}=0.245$). Follow-up analyses revealed that PE significantly decreased from day to night-time in controls (*t*_25_ = 9.070, *p* < 0.001, *r* = 0.876), MCS (*t*_16_ = 2.497, *p* = 0.024, *r* = 0.530), as well as by trend in UWS (*t*_17_ = 1.958, *p* = 0.067, *r* = 0.430) (Fig. 6).

The size of the observed effect was statistically larger in controls than in MCS (*z*_*diff*_ = 2.26, *p* = 0.012, one-tailed), as well as the UWS (*z*_*diff*_ = 2.71, *p* = 0.003, one-tailed) sample.

Supplementary analysis also revealed higher PE for periods with eyes open compared to eyes closed in a subset of patients (n = 22 and n = 10 during day and night-time, respectively) (cf. Suppl. Fig. 3). Furthermore, single-subject analysis indicate that more than half of the patients show preserved day to night variations of oscillatory brain activity and signal complexity with this effect being rather independent of their diagnosis (UWS or MCS) (cf. Suppl. Fig. 4).

PE calculations for frontal, central and parietal brain regions separately can be found in the Supplementary Material (Suppl. Fig. 5).

### Prognostic Value of Brain Dynamics

Lastly, we exploratively evaluated prognostic values of oscillatory brain dynamics in a subset of patients (n = 24). As predictors we used density of sleep spindles, density of slow waves, PE, alpha-to-theta ratio and high-to-low frequencies ratio. The subsequent follow-up diagnosis categories were considered: (i) death, (ii) UWS or lower severe disability (SD-), (iii) MCS or emergence from MCS (eMCS).

Multinomial logistic regression analysis revealed significance of our model (*χ* ^2^(10) = 32.849, p = 0.038, AIC = 56.849), which turned out to be a good fit to the data (deviance statistics not significant: *χ* ^2^(36) = 32.849, *p* = 0.619). Density of sleep spindles (*χ* ^2^(2) = 12.266, p = 0.002) and by trend also alpha-to-theta ratio (*χ* ^2^(2) = 5.031, p = 0.081) had a significant main effect on the follow-up diagnosis. The individual parameter estimates indicated, that density of sleep spindles significantly predicted whether a patient passed away or became MCS/MCS + (*Wald χ* ^2^(1) = 4.089, *e* ^*β*^ = 9957818, *p* = 0.043), and revealed a trend towards a significant prediction of whether patient passed away or became UWS/SD− (*Wald χ* ^2^(1) = 3.157, *e* ^*β*^ = 2452164, *p* = 0.076). In particular, the expected risk of a patient to pass away rather than survive (and become UWS/SD− or MCS/MCS+) is lower for subjects with higher density of sleep spindles. On the other hand, whether patients became UWS/SD− rather than MCS/eMCS was by trend predicted by permutation entropy (*Wald χ* ^2^(1) = 2.791, *e* ^*β*^ = 1.01^14^, *p* = 0.095); progressing to MCS/eMCS rather than to UWS/SD− is more likely for patients with more complex EEG signals.

## Discussion

In the present study we systematically focused on day-night variations of oscillatory EEG activity and sleep patterns in a large sample of UWS and MCS patients which were recorded over 24 hrs at bedside. We here explored diurnal day-night changes of brain dynamics with traditional measures used in sleep research, as well as a permutation entropy which is quantifying signal complexity and is known to be more resistant to artifacts. Interestingly we found that the prevalence of faster frequencies over slower frequencies decreased from day to night-time in controls and MCS patients only. Applying entropy measures we observed higher signal complexity during light-on periods as compared to night-time only in controls and MCS patients. Interestingly, these day-night differences of brain dynamics seem to systematically increase from UWS to MCS to control participants which mirror their cognitive abilities or degree of conscious awareness. Focusing on the two sleep patterns most characteristic for NREM sleep, namely sleep spindles and slow waves, we surprisingly did not find statistically significant differences between day and night in any of the patients groups. However we replicate earlier findings that (parietal) sleep spindles are linearly related to diagnosis/CRS-R scores or even outcome^14,15,17,18,20^.

The dominance of slow oscillatory brain activity informs about the synchronicity of cortical neurons and is usually associated with lower signal complexity or information in the signal. They usually hallmark states like deep sleep or pathological states with associated absence of conscious awareness. Previous research suggested UWS and MCS patients to differ in terms of spectral power, with more pronounced lower or higher frequencies in UWS and MCS patients, respectively^8,19,21–25^. Our results add to these findings by indicating that the reactivity of these EEG frequencies to a circadian Zeitgeber, namely light, is also different across clinical entities. Similarly, earlier findings of lower signal complexity in UWS compared to MCS patients^8,22,26,27^ were here extended by the observation that UWS patients do not even exhibit robust signal complexity changes across such extreme shifts as day and night. Diurnal changes in entropy across day and night are to be expected as daytime usually goes in hand with increased awareness and sensory processing. Conversely, night-time or unresponsiveness as in our patients should result in restricted information flow and lower signal complexity. Revealed day-to-night changes in power spectra and EEG signal complexity might reflect circadian fluctuations of arousal but remain to be tested explicitly. An additional single-subject analysis (cf. Suppl. Fig. 4) revealed that more than half of the patients show preserved diurnal variation of oscillatory brain activity and signal complexity, independently of the diagnosis. This means that on occasion even UWS patients may show day-night differences in entropy or slower oscillations. Composed ratios of frequency bands, like alpha-to-theta or high-to-low, are distinguishing well between day and night-time periods in controls and MCS patients, but on a group level only. For individual assessment these measures might be of little value as they do not allow distinguishing UWS from MCS patients. If systematic day to night variations in oscillatory activity or entropy have predictive relevance for outcome is yet to be investigated.

In healthy individuals sleep spindles and slow waves serve as robust markers for sleep and seem to have a crucial role in offline information processing or sleep consolidation. Only in healthy subjects we observed state-dependent (sleep vs. wake) changes in the number and length ratio of the slow waves. It is interesting to see why sleep-specific EEG patterns like spindles and slow-waves do not seem to distinguish between day and night periods in our UWS and MCS patients. One reason certainly can be the pathological changes of these patterns in topography, power, frequency (Hz) or morphology. Also it has to be noted that the used data for controls was acquired over a single stretch of 8 hours during night-time and does not encompass the entire spectrum of wakeful brain activity over night and day-time. We thereby however capture the brain’s state of wakefulness in recumbent position as seen in our patient group. A sample of hospitalized and recumbent subjects (and without medication) over 24 hours would be clearly advantageous as control population yet is difficult to realize.

Given the extensive analysis of our sleep data we believe that classical approaches like sleep classification according to criteria established in healthy individuals (AASM) or also traditional sleep pattern classification with unchanged parameters may often be misleading and would need to be tailored to the unique patterns observed in DOC EEG. Even when trained sleep scoring experts attempted classification of the patients’ recordings into only three broad states (WAKE, NREM and REM stages) we decided to abandon this approach due to inconsistent stagings and an inevitable lack of agreement. Ubiquitous pathological eye movements, spasms, general dysregulation of muscle tone, the much slower EEG spectrum and the usually absent alpha peaks, together with unusual topographies or simultaneous presence of hallmarks of different sleep stages in the same epoch rendered even a rough sleep-classification implausible in our opinion (cf. Suppl. Fig. 6).

A finding which is not straightforward to explain to-date is the change of symmetry of our detected slow-waves in controls and MCS patients. To our experience slow waves smaller than the 75 μV criterion are defined by longer down compared to up-states, and this asymmetry decreases with increasing peak-to-peak amplitude. Therefore we believe that the enhanced symmetry observed during light-off night periods (cf. Fig. 5) is related to the enhancement of slow-wave amplitudes and appears therefore under residual circadian control even in MCS patients. It should be noted that the attempt of introducing more strict criteria for slow wave detection (controlling for length and amplitude as for example in ref.^28^) rendered detections almost absent, even in healthy controls, which likely is related to the methodological limitation of using average-referencing (due to not correctable mastoid artifacts over 24 hrs periods) in the present study. It might also be of interest to further analyze morphological characteristics of spindles or slow-waves as well as cycling alternating patterns (CAPs) as parameters of this kind have been reported to be altered across various pathologies.

Preliminary evidence of circadian rhythmicity in DOC patients indicates deviations from the norm^29–32^. Our current protocol however precluded more fine-grained analysis than the diurnal day-night time variations reported herein. It is also possible that patients exhibit fluctuations with a shorter or longer periodicity^22^. This interesting issues regarding true circadian rhythmicity in DOC patients should be further explored with measures, which also can sample continuously across multiple days, such as core body temperature or melatonin assessments. Still, the present findings are the first of its kind and suggest that diurnal variations in brain activity can be identified using EEG in MCS (as well as some UWS patients). An important sanity check of such analyses can the actual state of the eyes, that is periods of eye opening vs. eye closure. Supplementary analysis performed on a subset of available patients suggests that EEG complexity is higher during eyes open versus eyes closed states in both clinical entities. Yet given the long periods of invisible eyes in our 24 hour recordings we can at present not reliably disentangle whether periods of eyes opening are indeed longer during the day as compared to the night in our patients (cf. Suppl. Fig. 3). Future studies addressing circadian questions in DOC patients should therefore verify that the state of the eyes can be recorded reliably over a full circadian cycle including bright day and dark night periods.

It is concluded that whereas traditional sleep staging and analysis seems hardly applicable using established criteria, we identified systematic day to night-time variations in spectral EEG and entropy measures. Interestingly, present data indicates that these diurnal day to night variations of brain dynamics are evident in healthy individuals and MCS patients with even UWS patients showing trends of similar direction. A good part of DOC patients therefore still seem to vary systematically across day-and-night and may thus indicate coupling to external “Zeitgebers” such as light. Given this, it is suggested that assessing residual cognitive abilities experimentally or the behavioural state of a DOC patient clinically, preferably occurs at times when the circadian system sends signals for waking or maximum arousal.

## Materials and Methods

For the purpose of assessing diurnal variations in oscillatory activity in different DOC states, we recorded continuous, long term (~24 h) polysomnography (PSG) in a sample of 40 patients as well as full-night (~8 h) PSG in a control population of 26 healthy participants.

Informed consent was obtained beforehand from all the healthy controls as well as from relatives or legal representatives of all the patients. The study was carried out in accordance with the ethical principles of the World Medical Association^33^. The experimental protocol was approved by the Ethics Committees of the Medical University of Graz and of the University of Salzburg.

### Participants

Forty patients in DOC states were recruited from clinics in Austria (n = 18) and in Belgium (n = 22). Diagnoses of the patients were established using behavioural evaluation of auditory, visual, motor, oromotor, communication and arousal functions according to Coma Recovery Scale-Revised criteria (CRS-R)^34,35^. Informed consent was obtained from relatives or legal representatives. Two patients with varying diagnosis and three patients who were exposed to constant light for 24 h were excluded from further analysis. The final sample therefore consisted of 18 patients in UWS (mean age = 45.2 ± 18.1 years, 11 males) and 17 patients in MCS (mean age = 45.9 ± 15.6 years, 13 males). For detailed information about the patients please refer to Table 1.

**Table 1:** Demographic patient data.

Nb	Age (years)	Sex	Aetiology	Time since injury (months)	Stage of disease	Clinical assessment	CRS-R Total score	Follow-up diagnosis	Time between recording and follow-up (months)
1	74	F	TBI	1	sub-acute	UWS	3	death	24
2	19	M	SSPE	24	chronic	UWS	3	—	—
3	65	F	anoxia	4	sub-acute	UWS	4	UWS	18
4	52	M	TBI	13	chronic	UWS	4	MCS	14
5	58	F	CVA	28	chronic	UWS	4	death	—
6	50	F	CVA	45	chronic	UWS	4	—	—
7	62	M	CVA	1	chronic	UWS	4	death	1
8	61	M	anoxia	32	chronic	UWS	4	death	—
9	46	M	anoxia	108	chronic	UWS	5	death	73
10	54	M	anoxia	9	chronic	UWS	5	death	10
11	37	M	anoxia	9	chronic	UWS	5	death	—
12	21	M	TBI	7	sub-acute	UWS	6	UWS	150
13	16	M	TBI	21	chronic	UWS	6	—	—
14	61	F	CVA	1	sub-acute	UWS	6	SD-	12
15	39	F	TBI	152	chronic	UWS	7	death	—
16	16	F	TBI	1	sub-acute	UWS	7	SD-	12
17	50	M	TBI	147	chronic	UWS	8	death	—
18	32	M	TBI	6	sub-acute	UWS	8	UWS	60
1	36	M	TBI	6	sub-acute	MCS	6	UWS	12
2	45	M	TBI	12	sub-acute	MCS	8	eMCS	24
3	62	M	TBI	2	sub-acute	MCS	8	—	—
4	34	M	anoxia	240	chronic	MCS	8	—	—
5	66	M	CVA	3	sub-acute	MCS	10	—	—
6	61	M	anoxia	2	sub-acute	MCS	10	—	—
7	48	M	TBI	8	sub-acute	MCS	11	death	—
8	31	F	CVA	1.5	sub-acute	MCS	11	—	—
9	57	M	anoxia	135	chronic	MCS	12	MCS	61
10	56	F	anoxia	85	chronic	MCS	12	MCS	67
11	21	M	anoxia	28	chronic	MCS	13	MCS	20
12	30	M	TBI	120	chronic	MCS	13	MCS	67
13	20	M	TBI	36	chronic	MCS	13	SD-	48
14	50	F	TBI	113	chronic	MCS	14	—	—
15	73	M	CVA	8	chronic	MCS	17	MCS	21
16	48	M	CVA	1.5	sub-acute	MCS	18	—	—
17	43	F	TBI	6	sub-acute	MCS	21	—	—

The analyzed patient sample consisted of 18 UWS and 17 MCS subjects. Abbreviations: Sex: M = male, F = female; Aetiology: TBI = Traumatic Brain Injury, CVA-Cerebrovascular Accident, SSPE = Subacute Sclerosing Panencephalitis; Clinical assessment and follow-up diagnosis: UWS = Unresponsive Wakefulness Syndrome, SD- = lower severe disability (3 points on Extended Glasgow Outcome Scale), MCS = Minimally Conscious State, eMCS = emergence from MCS; CRC-R = Coma Recovery Scale-Revised.

Follow-up on patients’ development was obtained by families and clinics. Participants, on whom the information was available, were assigned a follow-up diagnosis, according to the most recent CRS-R score or Extended Glasgow Outcome Scale score (GOSE)^36^: death, UWS (suitable CRS-R score or GOSE = 2), lower severe disability (SD-, GOSE = 3), MCS or eMCS.

In addition to the 35 patients we analysed data of 26 healthy controls (mean age = 35.0 ± 10.3 years, 13 males) which were previously recorded for a study investigating healthy and disturbed sleep in the general population (part of the data published here^37^).

### Data Acquisition and Pre-Processing

The PSGs of DOC patients were recorded with Brain Products amplifiers (Brain Products, Gilching, Germany). In the Austrian sample 18 EEG channels were distributed over the scalp according to the 10–20 system^38^: F3, Fz, F4, F7, F8, FC5, FC6, C3, Cz, C4, P3, Pz, P4, T3, T4, PO7, PO8 and Oz, online referenced to FCz. In the Belgian sample 12 EEG channels were utilized: F3, Fz, F4, C3, Cz, C4, P3, Pz, P4, T3, T4 and Oz, online referenced to the nose. The PSGs of healthy controls were recorded with Synamps EEG amplifiers (NeuroScan Inc., El Paso, Texas) using 21 EEG channels, online referenced to FCz. In addition to EEG, also electrocardiography (ECG), electromyography (EMG), electrooculography (EOG) and respiratory signals were recorded in all subjects. All data were recorded with a sampling rate of 500 Hz. In addition, continuous infrared video recordings could be obtained from 23 DOC patients. The average recording time in the DOC patients and healthy controls was 23.11 (±2.87), and 8.07 (±0.61), respectively.

PSG recordings in DOC patients, especially when performed over extended periods of time, are known to be affected by various artefacts. Dysregulation of the vegetative nervous system for example causes abnormal sweating, spasms result in huge muscle artefacts, and daily nursing activities as well as medical equipment introduce considerable amounts of noise. To account for those artefacts we introduced the following data pre-processing protocol, which was performed in Brain Vision Analyzer software (Brain Products GmbH, Gilching, Germany, version 2.0). The data was cut-off-filtered at 1 and 30 Hz with an infinite impulse response (IIR) butterworth filter (24 dB/oct slope). For the whole recording period the filtered data was then visually inspected for noisy channels (*first data inspection*) and re-referenced to the common reference composed of all noise-free scalp and mastoid channels. Conventional practise of re-referencing data to inactive mastoids channels had to be abandoned as the signal quality on these channels varied too much over our extended recording period of about 24 hours. Oculomotor artefacts were minimized using the regression-based technique of Gratton & Coles^39^, as on the basis of bipolar vertical and horizontal EOG channels. Lastly the data was visually inspected for remaining artefacts (*second data inspection*) and further processed.

If video recordings for the duration of the PSG recordings could be obtained, these were reviewed and all segments containing nursing behaviours (like cleaning, feeding, re-positioning, switching the lights on during the night), physiotherapy activities, family visits, or other disturbances in the patient’s room, were likewise removed during the *first data inspection*.

To explore day-night variations, we divided the long-term recordings into periods of lightsomeness, which corresponds to circadian day (named day-time), and periods of darkness, which corresponds to circadian night (named night-time). Day-time segments consisted of all artefact-free data between 8 am and 8 pm. Night-time segments included all clean data, when the lights in the room where switched off, between 11 pm and 5 am. Data between 8 pm and 11 pm was entirely excluded from the analysis to bypass periods at twilight. Furthermore, periods during night-time with lights turned on were rejected from further analysis. Data of three patients who were exposed to constant light for 24 h were altogether excluded from analyses.

On average, the length of day-time segments in UWS group was 6.9 h (±3.5 h) and in MCS group was 7.9 h (±3 h). Night-time segments lasted on average in UWS group for 5.1 h (±0.7 h) and in MCS group for 5.6 h (±0.4 h).

For comparison, overnight (~8 h) recordings of healthy control subjects were pre-processed in the same way and then were semi-automatically sleep staged according to criteria of American Academy of Sleep Medicine (AASM)^9^ by The Siesta Group© (Somnolyzer 24 × 7)^40^. Afterwards those nocturnal recordings of healthy subjects were divided into sleep period composed of N1, N2, N3 and REM sleep stages (analogously named “night-time”) and into wake periods composed of all non-sleep periods during time in bed (analogously termed “day-time” in the following).

### Oscillatory Brain Dynamics

For extracting the spectral power density, day-time and night-time segments were divided into 60 sec (artifact free) windows and Fast Fourier Transformation (FFT) was applied with 10% Hanning window. Spectral power was calculated as the averaged area information (μV × Hz) for three midline electrodes: Fz, Cz and Pz, and for four frequency bins: delta (2–4 Hz), theta (4–8 Hz), alpha (8–12 Hz) and beta (12–30 Hz). In order to obtain more robust interindividual power estimates we computed relative (divided by the total area information: 2–30 Hz) EEG power. We also utilized two ratios (high 8–30 Hz to low 1–8 Hz; as well as alpha to theta), which were previously found to be robustly associated with CRS-R scores in patients^19^.

With respect to characteristic graphoelements during sleep we analysed sleep spindles and slow waves, and compared their prevalence between the three groups (i.e. UWS, MCS and controls) and across the diurnal cycle (i.e. day-time and night-time).

Sleep spindles were detected on 6 scalp channels (F3, F4, C3, C4, P3, P4) using the following criteria: (i) frequency between 11 and 15 Hz, (ii) amplitude higher than 25 μV, (iii) duration longer than 0.5 second, (iv) controlled for muscle (30–40 Hz) and alpha (8–12 Hz) artefacts^41^. The automatic spindle-detection algorithm utilized was developed by The Siesta Group© (Somnolyzer 24 × 7) and is based on the features of 8124 spindle episodes visually identified by a group of experts (the ‘gold standard’) (for details see ref.^40^). Because often sleep spindles were detected only over one hemisphere, for each participant and each diurnal time separately we chose the hemisphere with highest total number of sleep spindle detections. Ultimately prevalence of sleep spindles was investigated for three cortical sites: frontal, central and parietal.

Slow waves were detected on 1–4 Hz filtered data recorded over frontal sites. For allowing robust slow waves detections (independent of eye artefacts and other artificial slow drifts) we pooled signals from those anterior channels (frontal and fronto-central electrodes), which were not excluded during the *data inspections*. Slow waves were then detected with in house-built Matlab routines (MathWorks®, Natick, MA) using criteria introduced by Riedner and colleagues^42^ requiring a negative zero crossing to precede a positive zero crossing in a time window between 0.25 to 1 seconds. To further investigate morphology of identified slow waves, we computed their peak-to-peak amplitude and ratio of the positive and negative lengths (measure of asymmetry).

Last but not least we computed PE of the EEG signal, which reflects its level of irregularity or unpredictability^43^. PE achieves its maximal value for highly complex or random signals. The minimal value of PE is 0 and can be attained by a continuously increasing or decreasing signal. However, an effective minimum for an EEG signal is about 0.4^44^ and indicates predominance of certain types of symbols or “low complexity” in the signal.

For analysis the pre-processed recordings were divided into 30 s-long epochs and PE was initially calculated for each epoch and across a set of electrodes (F3, Fz, F4, C3, Cz, C4, P3, Pz, P4, T3, T4 and Oz) separately. The embedding dimension was set to *n* = 3^44^ and lag parameter to *τ* = 3. Finally, PE values were averaged across epochs and electrodes, separately for the day and night-time segments.

### Statistical Analysis

Statistical analysis was performed with IBM® SPSS® Statistics (Version 23; SPSS Inc., Chicago, Illinois). To correct for multiple comparisons, post-hoc tests were adjusted with the False Discovery Rate (FDR) procedure^45^. Results are considered significant at *p* < 0.05 and denoted as trend at *p* < 0.1. Two-tailed tests were performed unless explicitly indicated otherwise. The statistical models were compared in terms of the Akaike Information Criterion (AIC), which takes into the account both the quality of the fit and the complexity of the model^46^.

Diurnal changes in EEG power were investigated with a series of mixed-design ANOVAs, with relative oscillatory band power as the dependent variable, DIURNAL TIME (day-time, night-time) as the within-subject factor and DIAGNOSIS (UWS, MCS, control) as the between-subject factor. Each cortical site (Fz, Cz and Pz) and each frequency band (delta, theta, alpha, beta) was considered separately. Significant day to night-time differences were post-hoc investigated with paired-sample tests.

Similarly, differences in entropy were evaluated with a mixed-design ANOVA, with DIURNAL TIME as within-subject factor, DIAGNOSIS as between-subject factor and averaged PE values as dependent variable.

Number of sleep spindles and slow waves were identified in day and night-time recordings. Firstly we modelled the relationships between the likelihood of occurrence of these oscillatory sleep patterns on the one hand, and DIAGNOSIS as well as DIURNAL TIME on the other hand. Since the distributions of the number of sleep spindles and slow waves were overdispersed, we used negative binomial models rather than Poisson models^47^. We performed a series of negative binomial linear regressions with log link function and DIAGNOSIS, DIURNAL TIME, as well as an interaction between the two as the predictors. Duration of the recording was used as an offset variable. The two types of oscillatory patterns (sleep spindles, slow waves) were investigated separately. In addition, we investigated the morphology of the detected slow waves (amplitude and length ratio) with a series of factorial ANOVAs, with DIAGNOSIS as a between subject factor and DIURNAL TIME as a within-subject factor. Furthermore we calculated Spearman’s correlation coefficients between the CRS-R total score and the density (i.e. number of detections normalized by the recording length) of oscillatory sleep patterns, for the entire clinical sample pooled together (N = 35).

Lastly we adopted a procedure similar to Arnaldi^48^ to exploratively evaluate prognostic value of oscillatory brain dynamics. We analysed a subset of patients whose later development was known (n = 24) with multinomial logistic regression. For the simplicity of the model, we divided the follow-up diagnoses into three categories: death, UWS/SD− and MCS/eMCS. As predictors we used density of sleep spindles, density of slow waves, PE, alpha-to-theta ratio and high-to-low frequency ratio, computed as means of the day and night values, and averaged over all cortical sites.

## Electronic supplementary material

Supplementary Information
