# Supplementary material for: Night and day variations of sleep in patients with disorders of consciousness
Source: Sci Rep. 2017 Mar 21;7:266. doi: 10.1038/s41598-017-00323-4 (PMC5428269; doi:10.1038/s41598-017-00323-4)
Supplement: Supplementary file 1 — Supplementary Information [file 41598_2017_323_MOESM1_ESM.pdf]

Night and day variations of sleep in patients with disorders of consciousness

- Supplementary materials -

Malgorzata Wislowska[1], Renata del Giudice[1], Julia Lechinger[1], Tomasz Wielek[1], Dominik P. J. Heib[1], Alain Pitiot[2], Gerald Pichler[3], Gabriele Michitsch[4], Johann Donis[4] and Manuel Schabus[1]*

[1]Laboratory for Sleep, Cognition and Consciousness, & Centre for Cognitive Neuroscience, University of Salzburg, Salzburg, Austria

[2]School of Psychology, University of Nottingham, Nottingham, United Kingdom

[3]Apallic Care Unit, Neurological Division, Albert-Schweitzer-Klinik, Graz, Austria

[4]Apallic Care Unit, Neurological Division, Pflegewohnhaus Donaustadt, Vienna, Austria

*corresponding author: Manuel Schabus, PhD, University of Salzburg, Hellbrunnerstr. 34, 5020 Salzburg, Austria; Fax: +43-8044-5113; e-mail: Manuel.Schabus@sbg.ac.at

Power Density Spectra

Supplementary Figure 1 depicts distribution of specific frequency band power over frontal and parietal midline cortical sites. The analyses revealed significant DIURNAL TIME × DIAGNOSIS interaction in all four frequency bands over all three cortical sites. Specifically, over the frontal midline cortical site for delta ($F_{2,56} = 16.636, p < 0.001, \eta_p^2 = .373$), theta ($F_{2,56} = 9.293, p < 0.001, \eta_p^2 = .249$), alpha ($F_{2,56} = 8.081, p = 0.001, \eta_p^2 = .224$) and beta ($F_{2,56} = 7.907, p = 0.001, \eta_p^2 = .220$), over central midline cortical site for delta ($F_{2,54} = 21.711, p < 0.001, \eta_p^2 = .446$), theta ($F_{2,54} = 16.014, p < 0.001, \eta_p^2 = .372$), alpha ($F_{2,54} = 21.405, p < 0.001, \eta_p^2 = .442$) and beta ($F_{2,54} = 7.220, p = 0.002, \eta_p^2 = .211$), as well as over parietal midline cortical site for delta ($F_{2,54} = 56.376, p < 0.001, \eta_p^2 = .511$), theta ($F_{2,54} = 22.964, p < 0.001, \eta_p^2 = .460$), alpha ($F_{2,54} = 23.977, p < 0.001, \eta_p^2 = .470$) and beta ($F_{2,54} = 7.266, p = 0.002, \eta_p^2 = .212$). The FDR-corrected post-hoc paired-sample tests revealed that in the control group the power of lower frequencies (delta and theta) always significantly increased from day to night-time, whereas power of higher frequencies (alpha and beta) always significantly dropped. Power density spectrum of MCS patients revealed similar pattern of circadian changes, however the day-night differences did not always reach the level of statistical significance.

Specifically, over the parietal midline electrode we observed significantly enlarged relative delta and theta power with simultaneously significantly decreased alpha and beta power during night compared to day-time in both MCS (delta: $t_{15} = 4.170, p = 0.003, r = .732$; theta: $t_{15} = 2.732, p = 0.015, r = .577$; alpha: $t_{15} = 3.083, p = 0.010, r = .623$; beta: $t_{15} = 3.681, p = 0.005, r = .689$) and controls (delta: $T = 2.0, Z = 4.407, p < 0.001, r = .611$; theta: $t_{25} = 11.615, p < 0.001, r = .919$; alpha: $t_{25} = 7.495, p < 0.001, r = .832$; beta: $t_{25} = 7.169, p < 0.001, r = .820$). The signal recorded from the frontal midline site showed significant day to night-time changes in the control group for all four frequency bands, with delta ($t_{25} = 11.816, p < 0.001, r = .920$) and theta ($t_{25} = 11.093, p < 0.001, r = .912$) decreasing, and alpha ($t_{25} = 7.171, p < 0.001, r = .820$) and beta ($t_{25} = 8.663, p < 0.001, r = .866$) increaseing from night to day-time. In MCS group we could observe on that cortical site similar day-to-night changes, which revealed trends towards statistical significance (delta: $t_{15} = 2.682, p = 0.068, r = .569$; theta: $t_{15} = 1.916, p = 0.099, r = .444$ and beta: $t_{15} = 2.153, p = 0.096, r = .486$). Over central cortical site controls revealed again significant decrease of alpha ($t_{25} = 7.171, p < 0.001, r = .820$) and beta ($t_{25} = 8.663, p < 0.001, r = .866$) power, accompanied by significant increase of delta ($t_{25} = 11.816, p < 0.001, r = .921$) and theta ($t_{25} = 11.093, p < 0.001, r = .912$) power from day to night-time. MCS patients revealed here only a trend towards significant decrease of delta band from day to night-time ($t_{16} = 2.617, p = 0.075, r = .547$). In UWS we could not observe any statistically significant circadian changes in

oscillatory power, except for diminished theta band power during the day compared to night-time over Cz ($T = 0, Z = 3.296, p = 0.003, r = .565$). All reported p-values are FDR-corrected.

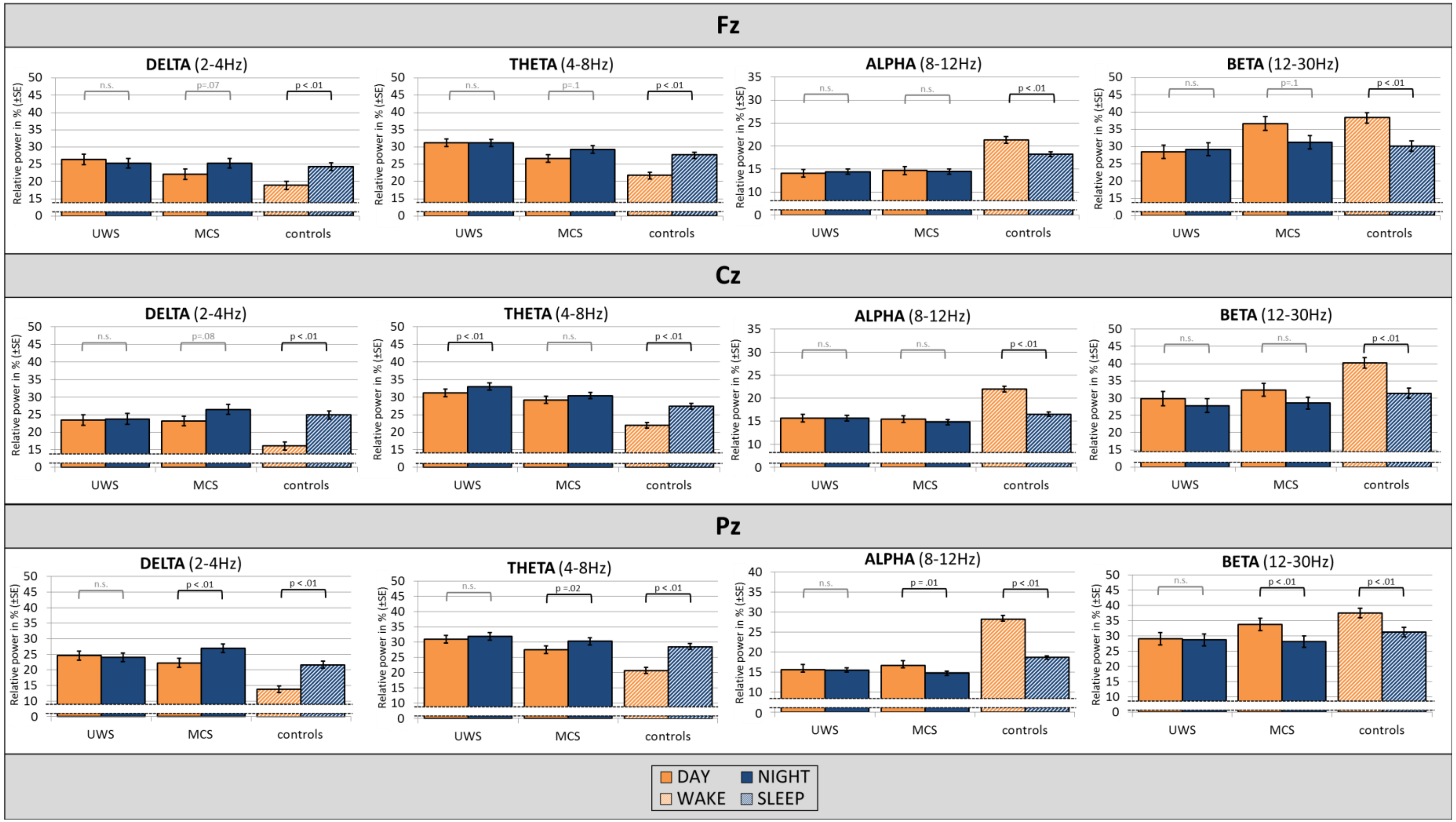

Fz
Cz
Pz
DELTA (2-4Hz)
THETA (4-8Hz)
ALPHA (8-12Hz)
BETA (12-30Hz)
Relative power in % (±SE)
UWS
MCS
controls
n.s.
p=.07
p=.1
p=.08
p=.02
p = .01
p < .01
DAY
NIGHT
WAKE
SLEEP

Supplementary Figure S1. Power spectra across day and night. In the MCS group day-to-night changes of oscillatory power resembled those observed in healthy control group, although the day-night differences were statistically significant in the MCS group only over parietal cortical site. No significant day-to-night changes were observed for the UWS group, except for theta band power over central electrode. Frequency-specific power values are normalized against total power (2-30Hz). Note the different scaling of the alpha power plot. P-values of post-hoc tests comparing day and night-time are highlighted for significant differences. Abbreviations: UWS=Unresponsive Wakefulness Syndrome; MCS=Minimally Conscious State.

Slow Waves

Supplementary Figure 2 depicts mean morphology and variance of the detected slow waves, averaged across all participants and all detections.

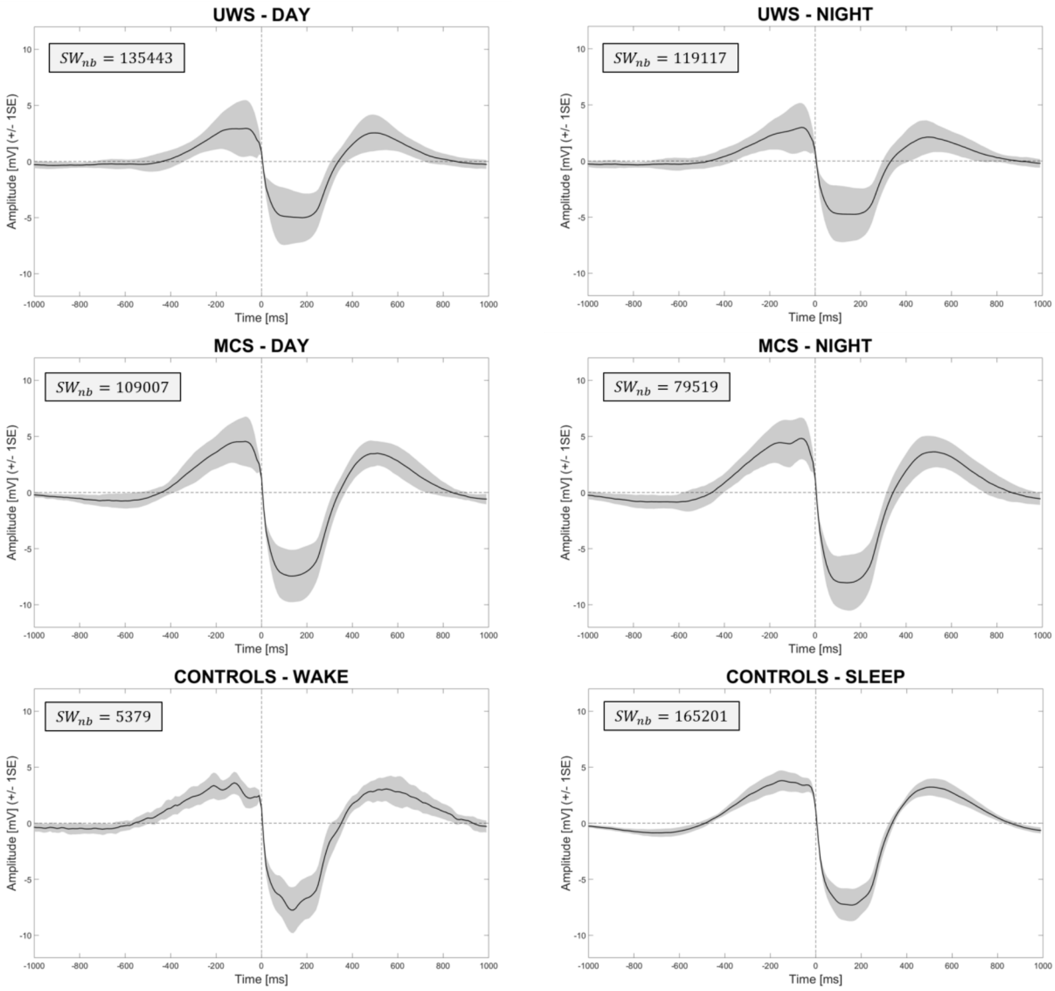

Supplementary Figure S2. Average morphology of the detected slow waves. The figure depicts slow waves averaged over detections and subjects. Separate plots are made for UWS (top panel), MCS (middle panel) and healthy control (bottom panel) group. The slow waves were detected from the pool of frontal electrodes. Note that for controls slow-waves are detected in 30sec epochs staged as wake or NREM sleep in an 8 hour nightly PSG recording. Grey area denotes +/- 1SE. In the top left corner the amount of averaged slow waves is provided. Data filtered between 1 and 4Hz. Abbreviations: SW_{nb}=number of Slow Waves; UWS=Unresponsive Wakefulness Syndrome; MCS=Minimally Conscious State.

PERMUTATION ENTROPY DURING EYES OPEN AND EYES CLOSED

Additionally we explored changes in brain activity measured with permutation entropy (PE) in relation to the behavioural state. We screened the available video recordings (n=23) of our patients and extracted periods of eyes opening and eyes closure. Unfortunately in many patients, especially during the night-time, the eyes were not visible. For the extracted data we calculated relative amount of segments with eyes open and eyes closed during the day and night-time, as well as the respective PE values. Paired-sample t-statistics revealed no significant changes from day to night-time in the amount of open vs. closed eyes. Analyses of PE revealed higher signal complexity for eyes open compared to eyes closed during day (UWS: $t_{11} = 3.914, p = .005, r = .763$; MCS: $t_9 = 2.838, p = .039, r = .687$) and night-time (UWS: $t_5 = 2.781, p = .039, r = .779$; MCS: $t_3 = 3.413, p = .042, r = .892$), irrespective of the diagnosis (suppl. Fig. 4). All p-values are FDR-corrected. Overall, both UWS and MCS patients show an increase in signal complexity when opening their eyes and this appears of similar magnitude for both diagnostic entities.

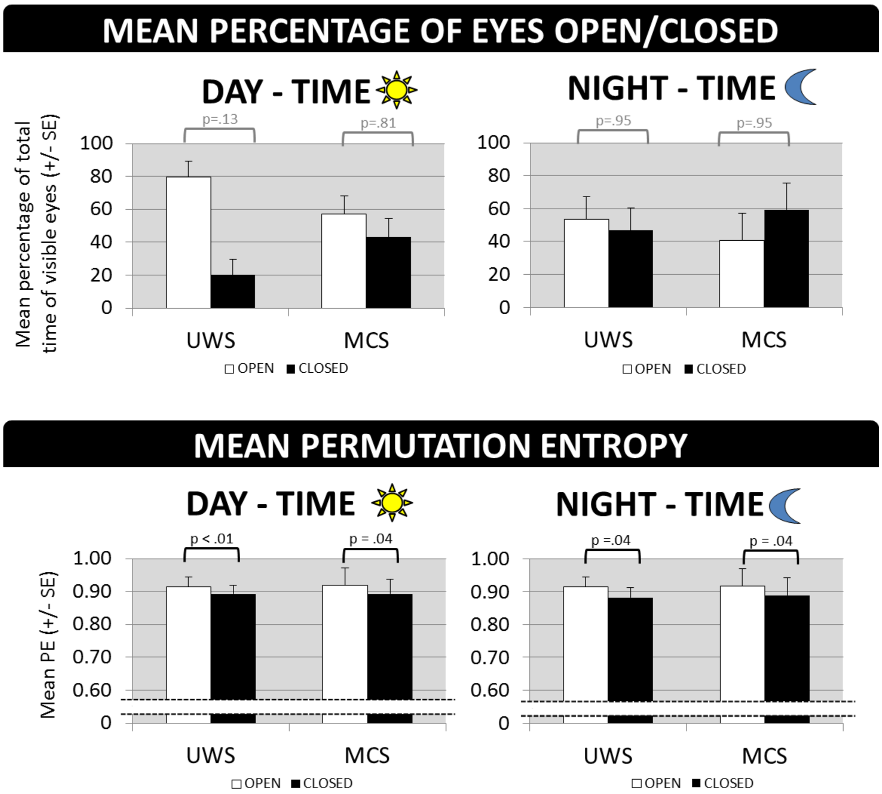

Supplementary Figure S3. Comparison of eyes open and eyes closed. Based on available video recordings, behavioral state of each patient was evaluated. Top panel: average amount of segments with eyes open and eyes closed was similar for each clinical group (UWS, MCS) and each diurnal time (day-time, night-time). Bottom panel: signal complexity was significantly lower during the periods with eyes closed compared to eyes open. All p-values are FRD-corrected. Abbreviations: PE, Permutation Entropy; UWS, Unresponsive Wakefulness Syndrome; MCS, Minimally Conscious State.

SINGLE SUBJECT ANALYSIS

We performed additional single-subject analysis of FFT (alpha-to-theta ratio and high-to-low frequency ratio) and of permutation entropy (PE). For each subjects separately, the three measures

were computed for every 30s-long segment. Subsequently, we compared with paired-sample t-tests the day and night-time values of each individual separately. The analyses revealed that while on the group level there seem to be statistical significant diurnal changes for MCS and not for UWS, on the individual level these differences appear more relative (suppl. Fig. 5). Ten out of 18 UWS patients and 10 out of 17 MCS patients had significantly higher alpha-to-theta ratio during the day compared to night-time; 4 UWS and 5 MCS show significant differences in the other direction. Eleven out of 18 UWS and 10/17 MCS had significantly increased high-to-low frequency ratio during the day compared to night-time; 5 UWS and 5 MCS show significant differences in the other direction. Lastly, 11/18 UWS and 12/17 MCS had significantly decreased PE from day to night-time with 4 UWS and 2 MCS showing differences in the other direction. These single subject comparisons highlight that on average more than half of the patients show preserved day to night variation of their brain activity irrespective of their diagnosis (UWS or MCS).

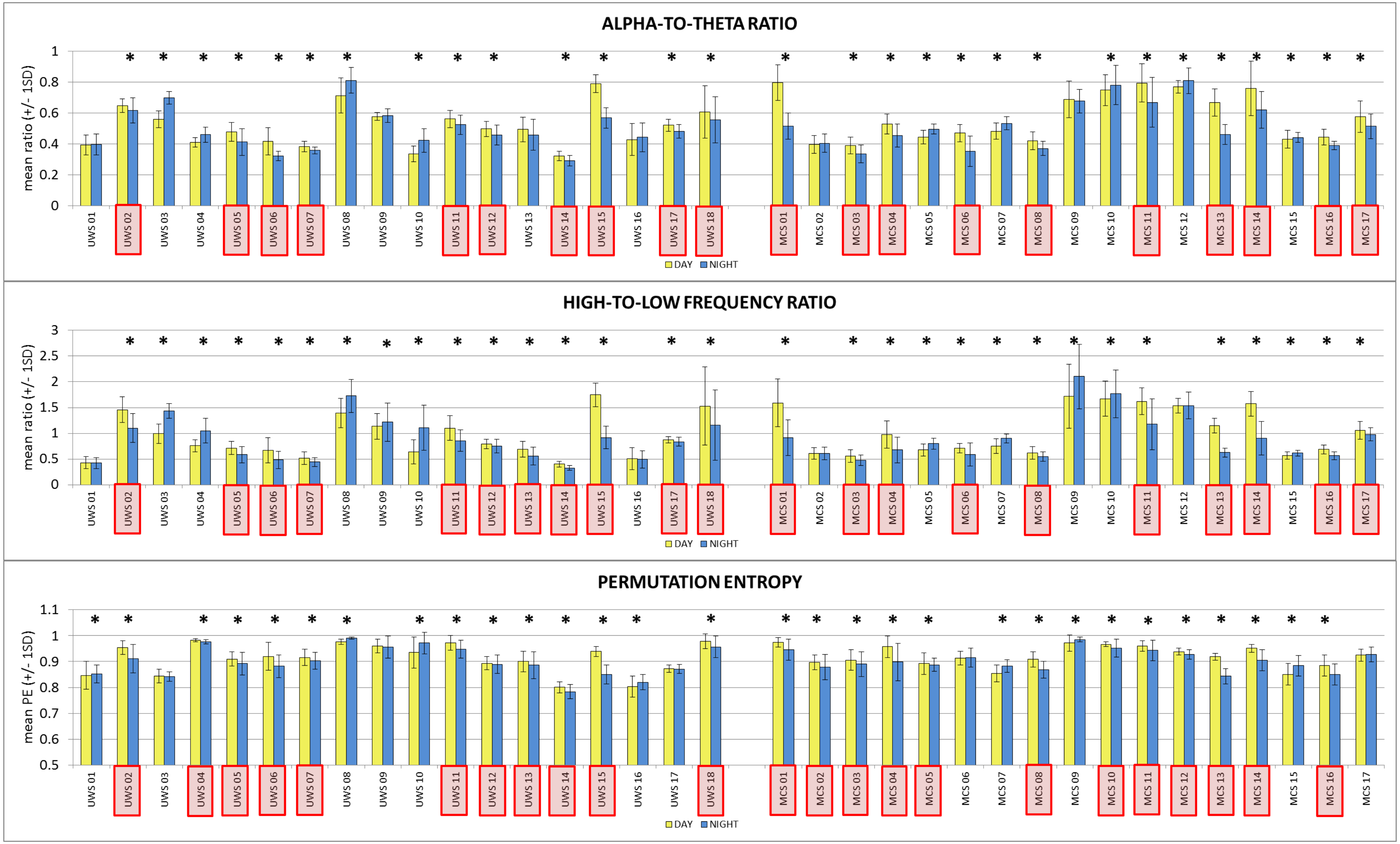
ALPHA-TO-THETA RATIO
mean ratio (+/- 1SD)
1
0.8
0.6
0.4
0.2
0
UWS 01
UWS 02
UWS 03
UWS 04
UWS 05
UWS 06
UWS 07
UWS 08
UWS 09
UWS 10
UWS 11
UWS 12
UWS 13
UWS 14
UWS 15
UWS 16
UWS 17
UWS 18
MCS 01
MCS 02
MCS 03
MCS 04
MCS 05
MCS 06
MCS 07
MCS 08
MCS 09
MCS 10
MCS 11
MCS 12
MCS 13
MCS 14
MCS 15
MCS 16
MCS 17
DAY
NIGHT
HIGH-TO-LOW FREQUENCY RATIO
mean ratio (+/- 1SD)
3
2.5
2
1.5
1
0.5
0
DAY
NIGHT
PERMUTATION ENTROPY
mean PE (+/- 1SD)
1.1
1
0.9
0.8
0.7
0.6
0.5
DAY
NIGHT

Supplementary Figure S4. Single subject analysis of oscillatory EEG activity as well as entropy. Mean alpha-to-theta ratio (top panel), mean high-to-low frequency ratio (middle panel) and mean permutation entropy (lower panel) calculated for each patient separately. Yellow and blue bars represent values averaged over all 30s-long segments, for day and night-time, respectively. Asterisks indicate all subjects with significant day-to-night changes, and red rectangles indicate subjects with significantly higher values for day compared to night-time (in the expected direction). The UWS and MCS subjects are in ascending order according to their total CRS-R score. Abbreviations: UWS, Unresponsive Wakefulness Syndrome; MCS, Minimally Conscious State.

Local Permutation Entropy

In addition to the permutation entropy (PE) calculated for the signal averaged across the entire brain, we also considered separately activity from the frontal, central and parietal midline cortical sites. Similarly to the computations performed for the entire brain, we divided here the signal from Fz, Cz and Pz into 30s-long epochs. PE was calculated for each epoch separately ($n = 3$ and $\tau = 3$) and then averaged across the time, for the two circadian times and three cortical derivations separately. For the statistical analysis we performed series of mixed-design ANOVAs with DIURNAL TIME (day-time, night-time) as the within-subject factor and DIAGNOSIS (UWS, MCS, controls) as the between-subject factor. Specific within-group differences between day and night-times were investigated with paired-sample tests, FDR-corrected for multiple comparisons, only when statistically significant interaction between DIAGNOSIS and DIURNAL TIME was observed. We found that DIAGNOSIS × DIURNAL TIME interaction was significant for frontal ($F_{2,56} = 8.188, p = 0.001, \eta_p^2 = .226$) and central ($F_{2,54} = 8.267, p = 0.001, \eta_p^2 = .234$) cortical sites. FDR-corrected post-hoc analyses revealed that complexity of the signal significantly dropped from day to night-time in MCS and UWS group over Cz (MCS: $t_{16} = 3.252, p = .015, r = .631$; UWS: $t_{13} = 2.212, p = .039, r = .572$), and by trend also over Fz (MCS: $t_{15} = 1.784, p = .095, r = .418$; UWS: $t_{15} = 2.100, p = .052, r = .465$), whereas in controls we observed a significant day-to-night decrease over both electrodes (Fz: $t_{25} = 9.182, p < .001, r = .878$; Cz: $T = 3.0, Z = 4.38, p < .001, r = .730$) (Supp. Fig. 2). The decrease of permutation entropy from day to night-time over Fz had significantly stronger effect in control than in MCS ($z_{diff} = 2.66, p = .004$, one-tailed) as well as in control compared to UWS ($z_{diff} = 2.55, p = .005$, one-tailed).

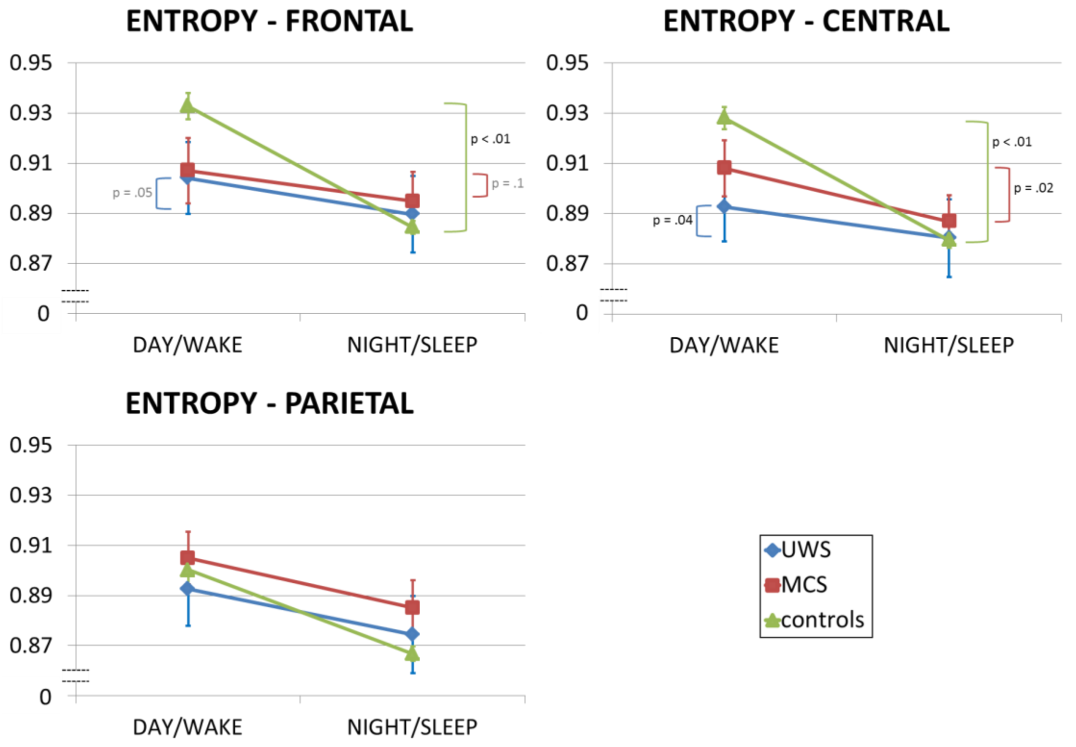

Supplementary Figure S5. Permutation entropy calculated for Fz, Cz and Pz. Over Fz and Cz the complexity of signal significantly dropped from day to night-time in control and MCS groups, but not in UWS group. For Pz statistically significant day to night-time difference was observed. It is to be noted that given the varying sites of brain lesions across clinical entities (in UWS as well as in MCS) a functional interpretation of the various topographies may be misleading. Abbreviations: UWS=Unresponsive Wakefulness Syndrome; MCS=Minimally Conscious State.

Sleep Staging

Supplementary Figure 3 depicts an exemplary 30sec time window of continuous EEG signal. Since sleep staging is based on such 30sec-epochs, we considered occurring waveforms.

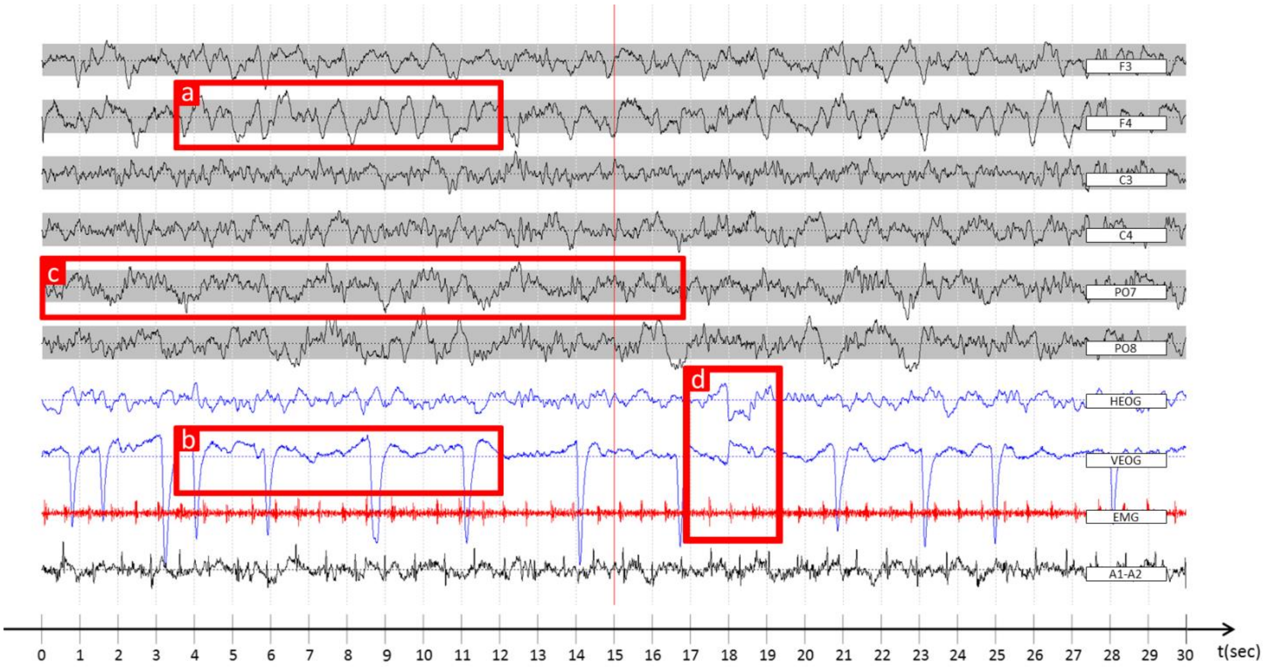

Supplementary Figure S6. Problems with DOC patients' sleep staging. The figure depicts exemplary 30sec epoch of PSG signal. Graphoelements present in this time window (indicated by small roman letters written in the white font) seem to be hallmarks of different sleep stages. However it is not certain that observed here waveforms correspond to those produced by healthy brains.

For example **(a)** low frequency oscillations predominantly occurring over frontal regions could be interpreted as slow waves and thus the epoch would be assigned sleep stage N3 (deep sleep); however **(b)** ubiquitous blinks present on VEOG don't normally occur during deep sleep, and are indicative for sleep stage W (wake) or possibly REM. It is also interesting to note that **(c)** background EEG activity over parieto-occipital sites (~0.5Hz) is of much lower frequency than the background EEG activity over frontal regions (~1Hz). Lastly **(d)** we can observe rapid eye movement concurring with the low chin EMG tone (here EMG is at the lowest level of the entire recording), which is typical for phasic REM sleep.

Exemplary data extraced from a day-time segment of one of MCS patient. Signal is preprocessed based on AASM ver.2.0 criteria[9]: EEG filtered 0.3-35Hz and EMG filtered 10-100Hz (+notch at 50Hz). EEG re-referenced to contralateral mastoids (A1 or A2). Color-coded PSG signal represents: black = EEG, blue = bipolar EOG and red = bipolar EMG. Grey bars behind EEG time-series have width of 75μV. While amplitudes of EEG and EMG signals are scaled the same, EOG amplitudes were down-scaled for the display purpose. Abbreviations: PSG, polysomnography; EEG, electroencephalography; EOG, electgrooculography; EMG, electromyography; HEOG, horizontal EOG; VEOG, vertical EOG; DOC, disorders of consciousness; MCS, minimally conscious state.
